# BACK‐on‐LINE^™^ : A Digital Pain Phenotyping Tool to Personalise Early Self‐Management of Low Back Pain: Reliability and Validity in Working Adults

**DOI:** 10.1002/ejp.70268

**Published:** 2026-04-11

**Authors:** Minghao Chen, Valerie Sparkes, Liba Sheeran

**Affiliations:** ^1^ School of Healthcare Sciences, Ty Dewi Sant, Heath Campus Cardiff University Cardiff UK; ^2^ School of Acupuncture‐Moxibustion and Tuina & School of Health Preservation and Rehabilitation Nanjing University of Chinese Medicine Nanjing China; ^3^ Biomechanics and Bioengineering Research Centre Versus Arthritis, Cardiff University Cardiff UK; ^4^ School of Health Sciences, Highfield Campus University of Southampton Southampton UK

**Keywords:** BACK‐on‐LINE, low back pain, nociceptive pain, nociplastic pain, reliability, self‐management, validity, workplace health

## Abstract

**Background and Objective:**

Low back pain (LBP) is a leading cause of work‐related disability. Although early tailored self‐management is advocated, current approaches remain generic. Classifying LBP by predominant pain phenotype (nociceptive, neuropathic, nociplastic) may enable targeted self‐management, yet practical tools for early or workplace use are limited. This study evaluated the reliability and validity of BACK‐on‐LINE, a self‐administered digital tool differentiating nociceptive and nociplastic pain to support early mechanism‐informed self‐management in working populations, benchmarked against established patient‐reported outcome measures (PROMs).

**Methods:**

Employed adults with LBP (*n* = 211) recruited from occupational settings completed BACK‐on‐LINE and PROMs assessing pain (Numeric Pain Rating Scale; NPRS), disability (Roland–Morris Disability Questionnaire; RMDQ), and psychosocial risk (STarT Back Screening Tool; SBST). Reliability was assessed using internal consistency (Cronbach's *α*) and test–retest reliability (intraclass correlation coefficient; ICC). Validity was examined through convergent validity with PROMs, known‐groups validity comparing nociceptive and nociplastic subgroups, and criterion validity using receiver operating characteristic (ROC) analyses against reference standards (pain intensity, disability, chronicity, sickness absence).

**Results:**

Data from 136 participants (64% completion) were analysed. BACK‐on‐LINE classified 68.4% as nociceptive and 31.6% as nociplastic. Reliability was strong (*α* = 0.83; ICC = 0.88). Convergent validity was moderate, strongest with SBST (*r* = 0.67). Known‐groups validity was robust, with nociplastic participants reporting higher pain, disability and psychosocial risk (all *p* < 0.001). Criterion validity was moderate (AUC = 0.67–0.77), with BACK‐on‐LINE demonstrating comparable or superior discrimination to SBST.

**Conclusions:**

BACK‐on‐LINE shows good reliability and multi‐dimensional validity for self‐classifying pain phenotypes in working adults with LBP, offering a scalable approach to support early mechanism‐informed self‐management in occupational health pathways.

**Significance Statement:**

This study provides robust initial evidence for a self‐administered digital tool BACK‐on‐LINE that enables working adults to self‐classify their low back pain into dominant pain mechanism phenotype. By supporting earlier, pain mechanism‐informed self‐management, it addresses a key gap in workplace back pain support, which is often generic advice poorly tailored. The tool's strength lies in scalability, early application and workplace relevance, outperforming a widely used prognostic tool in this context.

**Abbreviations**
AUC: area under the curve
CCC: clinical classification checklist
CI: confidence interval
HCRW: Health and Care Research Wales
IASP: International Association for the Study of Pain
ICC: intraclass correlation coefficient
LBP: low back pain
NICE: National Institute for Health and Care Excellence
NPRS: Numeric Pain Rating Scale
ODI: Oswestry Disability Index
PB: pain behaviour
PL: impact of low back pain on lifestyle
PP: pain perception
PW: impact of low back pain on work
QST: quantitative sensory testing
RMDQ: Roland Morris Disability Questionnaire
ROC: Receiver Operating Characteristic
SA: sickness absence
SBST: STarT Back Screening Tool
SD: standard deviation

## 1 Introduction

Low back pain (LBP) is the leading global cause of disability, affecting an estimated 619 million people in 2020 and projected to rise to 843 million by 2050—a 36% increase—despite nearly 40% of its burden being attributable to modifiable risk factors (Ferreira et al. 2023). The impact is particularly pronounced in working populations, where LBP contributes substantially to reduced work ability, sickness absence and productivity loss. Occupational prevalence varies widely across industries (1.4%–15.6%), reflecting heterogeneity in physical, organisational and psychosocial demands (Fatoye et al. 2019).

Clinical guidelines consistently advocate early self‐management as central to LBP care (NICE 2016), with education and activity promotion highlighted as core components of a multifactorial self‐management supporting sustained behavioural change (WHO 2023). However, many existing self‐management interventions show only modest and short‐term benefits, in part due to continued reliance on generic models that fail to account for the marked heterogeneity of LBP and underlying pain mechanisms (Nicholl et al. 2017; Scholz et al. 2024; Chen et al. 2025). In contrast, mechanism‐informed and individually tailored approaches are increasingly associated with improved outcomes and engagement, particularly within digital health interventions (Wang et al. 2024; Mason et al. 2024).

Pain phenotype classification is grounded in the biopsychosocial model and distinguishes three broad pain phenotypes: nociceptive pain (arising from actual or threatened non‐neural tissue damage), neuropathic pain (resulting from nerve damage) and nociplastic pain (arising from altered pain processing without clear tissue damage) (Nijs et al. 2024). Although these phenotypes are not diagnostic and may overlap, particularly through processes such as central sensitisation, identifying the dominant pain phenotype is clinically meaningful for shaping expectations, guiding treatment selection and supporting appropriate self‐management (Chimenti et al. 2018; Nijs et al. 2024). Despite advances in pain phenotyping (Smart et al. 2012; Nijs et al. 2023), the complexity and subjectivity of pain limit standardised clinical classification, and advanced assessment techniques remain impractical for routine use (Cao et al. 2024). Clinical practice therefore relies on brief screening tools such as the STarT Back Screening Tool (SBST), which stratifies risk of persistent disability, and painDETECT or Douleur Neuropathique 4, which screen for neuropathic features (Bouhassira et al. 2005; Hill et al. 2011; Bittencourt et al. 2022). While psychometrically robust, these tools are typically clinician‐administered, designed for single time‐point assessment, and poorly suited to symptom fluctuation or timely, personalised workplace management. Similarly, pain mechanism classification checklists require in‐person assessment and expert judgement, limiting scalability and accessibility (Smart et al. 2010, 2011).

To address these limitations, BACK‐on‐LINE (Data S1) was developed through international Delphi consensus as a self‐administered digital tool to support identification of predominant pain phenotypes in people with LBP (Alothman et al. 2018). The tool classifies individuals with LBP according to dominant pain phenotype, using clusters of clinical features including pain characteristics, functional impact, psychosocial context and behavioural patterns (Smart et al. 2010, 2011). The current prototype differentiates nociceptive and nociplastic pain, together accounting for most non‐specific LBP, while currently excluding neuropathic pain features that typically require specialist assessment (Bates et al. 2019; Kerckhove et al. 2021). Designed for integration within digital self‐management platforms, BACK‐on‐LINE, accessed following the initial occupational health encounter, provides immediate, tailored feedback aligned to the identified pain phenotype and enables individuals to access their pain‐phenotype specific self‐management digitally, supporting proportionate, timely and tailored workplace interventions.

Preliminary testing demonstrated high test–retest reliability and good construct validity in a clinical sample of individuals with LBP (Alothman et al. 2019). This study therefore evaluated the reliability and validity of BACK‐on‐LINE stratifying dominant pain phenotypes (nociceptive vs. nociplastic) in working adults with LBP in workplace settings. Reliability was assessed using internal consistency to examine how well the items work together and test–retest reliability to assess the stability of self‐classification over time. Validity was assessed using three complementary approaches aligned with the tool's purpose: convergent validity with PROMs to confirm expected associations with pain, disability and psychosocial risk; known‐groups validity through comparison of PROM scores between nociceptive and nociplastic subgroups to assess discrimination between clinically meaningful phenotypes; and criterion validity to evaluate classification performance against predefined clinical reference standards (pain intensity, disability, pain chronicity and sickness absence).

## 2 Methods

### 2.1 Study Design

This study used data from a prospective, single‐arm cohort study (Dec 2019–Oct 2022) evaluating the feasibility and acceptability of a digital health intervention incorporating BACK‐on‐LINE to support self‐management of LBP in workplace settings. Data were taken from baseline and a 4‐week follow‐up assessments.

### 2.2 Participants

Participants were working adults recruited from UK workplace settings in healthcare (NHS), transport and higher education. Eligible participants were adults (≥ 18 years), employed full‐ or part‐time or self‐employed with self‐reported LBP affecting their ability to work but still working, internet access and English proficiency. Participants were excluded if they: (1) were aged < 18 years; (2) reported clinical indicators of potentially serious spinal pathology or systemic illness, screened during recruitment using questions recommended by National Institute for Health and Care Excellence (NICE) clinical guidelines as indicators of serious spinal pathology; (3) were pregnant or breastfeeding; or (4) were involved in any other back pain research. Screening was conducted by an expert physiotherapist (LS) over a telephone prior to enrolment to ensure suitability for self‐care. Participants were excluded and referred to general practitioner if they answered ‘yes’ to one or more of the questions listed in Table 1.

**TABLE 1:** Screening questions for clinical indicators of serious pathology or systemic illness (based on NICE 2016 guidance).

No.	Screening question
1	Is your low back pain constant and worsening over the past 4 weeks?
2	Did your low back pain start or worsen significantly following a fall?
3	Did your low back pain coincide with feeling unwell (e.g., fever, chills, night sweats) without another explanation?
4	Do you have altered or loss of sensation around your back passage or genitals (e.g., noticeable when wiping after using the toilet)?
5	Are you experiencing any unexplained widespread weakness in one or both legs?
6	Do you have difficulty passing or controlling urine or faeces?
7	Did your low back pain coincide with unexplained trouble walking (e.g., limping, tripping, falling or feeling unsteady on your feet)?

### 2.3 Data Collection

After providing informed consent, eligible participants received an email with a secure weblink and a unique eight‐digit login code to access BACK‐on‐LINE and an online data collection system. Using this code, they completed a standardised questionnaire at baseline and after the 4‐week intervention. The questionnaire captured demographic and work‐related details (age, gender, education, employment status, job role and pain duration), the BACK‐on‐LINE and a validated patient‐reported outcome measure (PROMs). To determine eligibility for test–retest reliability analysis, the follow‐up survey asked, ‘Is your back pain better, same, or worse?’. Responses were dichotomised as recovered or not recovered to assess stability for inclusion.

Data security was ensured through password protection, user‐specific authentication and strict adherence to institutional and General Data Protection Regulation (GDPR) standards. Identifiable information was stored separately from research data and linked only via the unique identifier, ensuring anonymisation. All data were encrypted and securely stored on Cardiff University's Advanced Research Computing (ARCCA) infrastructure.

The cohort study received ethical approval from the University's Research Ethics Committee and Health and Care Research Wales (HCRW) Ethics Committee (19/HCRW/0035). All procedures complied with GDPR and participant consent agreements to ensure confidentiality and secure data management and dissemination of this study results.

### 2.4 Outcomes

#### 2.4.1 BACK‐on‐LINE

BACK‐on‐LINE is a 34‐item self‐administered mechanism‐oriented phenotyping tool designed to differentiate nociceptive from nociplastic pain presentations (Data S1) covering four domains: (i) Pain Behaviour (PB) (18 items) assessing pain type, duration, location and intensity, and pain‐related behaviours; (ii) Impact of LBP on Work (PW) (six items) evaluating how back pain affects work tasks; (iii) Impact of LBP on Lifestyle Social & Family Life (PL) (three items) examining effects on daily activities and family life; and (iv) Personal perceptions of LBP (PP) (seven items) exploring cognitive and affective factors including beliefs, attitudes and self‐efficacy in managing LBP.

Exploratory factor analysis (EFA) was conducted during preliminary validation to explore the conceptual interrelatedness of behavioural and perceptual constructs (Alothman et al. 2019). The analysis supported a multidimensional structure consistent with the predefined domains: Pain Behaviour, Impact on Work, Impact on Life and Pain Perception, with items loaded onto expected factors without cross‐loadings, supporting construct validity. Reliability analysis demonstrated corrected item‐total correlations of 0.22–0.75 for the overall BOL questionnaire and 0.16–0.77 within the Pain Behaviour domain. Cronbach's alpha if item deleted remained stable (0.86–0.87 overall; 0.78–0.80 for PB), indicating stable internal consistency without evidence of excessive redundancy (Alothman et al. 2019).

Each item is assigned a weighted score (Data S1) reflecting behavioural or perceptual indicators of dominant nociceptive or nociplastic pain phenotype, in accordance with The Low Back Pain Phenotyping (BACPAP) consortium's international and multidisciplinary consensus recommendations (Nijs et al. 2024) (Data S2).

The total score is calculated by summing all item scores, yielding a possible range of 2–136. Dominant pain phenotype is assigned using an empirically derived cut‐off score of 36, where scores ≤ 36 indicate nociceptive pain and scores > 36 indicate nociplastic pain.

This threshold was established in a prior validation study using receiver operating characteristic (ROC) curve analysis, with the high‐risk category of the STarT Back Screening Tool as the reference standard for nociplastic pain (Chen et al. 2023). The optimal cut‐off maximised the Youden index (0.613), balancing sensitivity (0.889) and specificity (0.724) for discriminating nociplastic from nociceptive pain.

#### 2.4.2 Patient‐Reported Outcome Measures (PROMs) Used for Validity Testing

To evaluate BACK‐on‐LINE's validity in classifying dominant pain mechanisms (nociceptive vs. nociplastic), we selected well‐validated instruments commonly used in low back pain (LBP) research and clinical practice. These were chosen to align with the International Classification of Functioning, Disability and Health (ICF) framework (WHO 2001), ensuring coverage of key domains: pain intensity (impairment), physical function (activity limitation) and psychosocial risk (contextual/personal factors). These constructs are also theoretically expected to differ between pain mechanisms, with nociplastic pain typically associated with greater pain, disability and psychosocial complexity (Nijs et al. 2024; Smart et al. 2012).

The Numeric Pain Rating Scale (NPRS), Roland–Morris Disability Questionnaire (RMDQ), and STarT Back Screening Tool (SBST) were used as external comparators to assess convergent, known‐groups and criterion validity. Cut‐off scores were applied only to support validity testing, not to define pain mechanism subgroups. For the SBST, the validated ≥ 4 cut‐off was used to identify higher psychosocial risk (Hill et al. 2008). For the NPRS, pain intensity was categorised as mild (≤ 3), moderate (4–6) or severe (≥ 7) (Boonstra et al. 2016). No fixed threshold was applied for the RMDQ; instead, we hypothesised that participants classified as having a nociplastic pattern would report higher disability. These cut‐offs reflect commonly accepted clinical categories.

- STarT Back Screening Tool (SBST): A 9‐item tool assessing physical and psychosocial risk factors, including fear, depression, catastrophising, worry and anxiety. The SBST has demonstrated excellent reliability (ICC = 0.90), good internal consistency (Cronbach's *α* = 0.73), and strong convergent validity with other LBP measures (*r* = 0.74) (Bruyère et al. 2014; Fuhro et al. 2016).
- Numeric Pain Rating Scale (NPRS): An 11‐point scale (0 = no pain, 10 = worst imaginable pain) for measuring pain intensity. It demonstrates excellent test–retest reliability (ICC = 0.95–0.97) (Alghadir et al. 2018).
- Roland–Morris Disability Questionnaire (RMDQ): A 24‐item measure of LBP‐related disability (range 0–24), with higher scores indicating greater functional limitation. The RMDQ has excellent test–retest reliability (ICC = 0.91) and strong internal consistency (Cronbach's *α* = 0.89) (Stratford and Riddle 2016; Jenks et al. 2022).

### 2.5 Statistical Analyses

Baseline characteristics were summarised using descriptive statistics with quantitative variables expressed as mean and standard deviation (SD), and categorical variables as counts, numbers and percentages. All analyses were performed using the Stata Statistical Software (version 14, Stata Corp LLC) with a significance level of 5%.

#### 2.5.1 Reliability

Internal consistency was assessed at baseline using Cronbach's alpha (*α*) inter‐item correlations, with adequacy based on classical test theory benchmarks: > 0.90 excellent, 0.80–0.90 strong, 0.70–0.80 moderate and < 0.70 weak (George 2011). Pearson's correlation coefficients were used to assess domain‐total correlations and interpreted as weak (*r* < 0.25), moderate (*r* = 0.26–0.50), strong (*r* = 0.51–0.74) or very strong (*r* ≥ 0.75) (Portney and Watkins 2009). Test–retest reliability was calculated using baseline and four‐week follow‐up data from participants who reported no change in LBP status; those reporting improvement or worsening excluded to ensure clinical stability and avoid confounding reliability estimates with true symptom change. Intraclass correlation coefficients (ICCs) with 95% confidence intervals (CIs) were computed using a two‐way mixed‐effects model (absolute‐agreement, single‐measure) with ICC values classified as low (0.21–0.40), moderate (0.41–0.60), high (0.61–0.80) or excellent (≥ 0.81) (Dubé et al. 2021).

#### 2.5.2 Validity

Convergent validity was assessed by examining correlations between BACK‐on‐LINE subdomain scores (PB, PW, PL, PP) and total scores with SBST for psychosocial risk (Hill et al. 2008), NPRS for pain intensity (Kahl and Cleland 2005) and RMDQ for functional disability (Roland and Morris 1983). Spearman's rank correlation coefficients were used and interpreted as weak (*r* < 0.25), moderate (*r* = 0.26–0.50), strong (*r* = 0.51–0.74) or excellent (*r* ≥ 0.75) (Portney and Watkins 2009).

Known‐groups validity was tested by comparing PROM scores between BACK‐on‐LINE subgroups classified as nociceptive or nociplastic. Continuous outcomes (RMDQ and BACK‐on‐LINE scores) were analysed using independent *t*‐tests or non‐parametric equivalents depending on distribution. Where PROMs were grouped into clinical categories (NPRS: mild/moderate/severe; SBST: risk categories), chi‐squared (*χ* ^2^) tests were used to assess whether groupings aligned with expected subgroup classifications. Effect sizes were calculated using Cohen's *d* for mean differences (small: 0.2–0.5; moderate: 0.5–0.8; large: > 0.8) (Eton et al. 2020), and Cramer's *V* for categorical outcomes (small: 0.07–0.21; moderate: 0.21–0.35; high: > 0.35, df = 2) (Kim 2017).

Criterion validity was examined by testing whether BACK‐on‐LINE subgroup classifications aligned with external reference criteria for disabling LBP. These included NPRS ≥ 7 for high pain intensity (Boonstra et al. 2016); RMDQ ≥ 14 for high disability (Roland and Morris 1983); SBST ≥ 4 for high prognostic risk (Hill et al. 2008); and ≥ 4 weeks of sickness absence (Côté et al. 2008). Discriminative ability was quantified using area under the receiver operating characteristic curve (AUC) with 95% confidence interval. AUC values were interpreted as low (0.60–0.69), moderate (0.70–0.79) or high (≥ 0.80) (Mandrekar 2010). BACK‐on‐LINE discrimination performance was compared to the SBST using DeLong's non‐parametric test.

## 3 Results

### 3.1 Characteristics of Participants

Data from 136 participants (36 males, 100 females; mean age = 42.6 years, SD = 10.0) who completed all measures were included in the analysis. Of these, 21 participants who also completed the BACK‐on‐LINE at the 4‐week follow‐up and reported no change in their LBP status were included in the test–retest reliability assessment.

Participants reported a wide range of pain durations, from a few days to over 6 months, and varying periods without pain. About half had never taken sickness absence due to LBP, and the majority (over 90%) remained in the same job role despite their pain.

Baseline participant characteristics and instrument scores are presented in Table 2.

**TABLE 2:** Baseline participant characteristics, BACK‐on‐LINE scores and patient‐reported outcomes (PROMs).

Characteristics	Value
**Pain duration, *n* (%)**	
0–7 days	25 (18.4)
8–14 days	15 (11.0)
15 days to 1 month	10 (7.3)
1–3 months	22 (16.2)
3–6 months	14 (10.3)
Over 6 months	50 (36.8)
**Pain‐free period, *n* (%)**	
0–3 months	43 (31.6)
4–6 months	14 (10.3)
7–12 months	18 (13.3)
1–10 years	40 (29.4)
Over 10 years	21 (15.4)
**Role change linked to LBP, *n* (%)**	
Remained in the same role	123 (90.5)
Moved to a new role	9 (6.6)
Left job but able to return	1 (0.7)
Part time work or temporary leave	3 (2.2)
**Sickness absence due to LBP, *n* (%)**	
Never had time off	71 (52.2)
Less than 1 week	22 (16.2)
1–4 weeks	22 (16.2)
4–8 weeks	10 (7.4)
2–6 months	8 (5.8)
More than 6 months	3 (2.2)
**BACK‐on‐LINE scores, mean (SD)**	
Total BACK‐on‐LINE score	31.9 (12.6)
Pain Behaviour (PB)	19.8 (9.2)
Impact on Work (PW)	2.1 (1.5)
Impact on Life (PL)	2.8 (2.1)
Perception of LBP (PP)	7.2 (2.7)
**RMDQ, mean (SD)**	6.2 (5.0)
**NPRS categories, *n* (%)**	
0–3	38 (27.9)
4–6	67 (49.3)
7–10	31 (22.8)
**SBST categories, *n* (%)**	
Low risk	85 (62.5)
Medium risk	42 (30.9)
High risk	9 (6.6)

Abbreviations: NPRS, Numeric Pain Rating Scale; RMDQ, Roland‐Morris Disability Questionnaire; SBST, STarT Back Screening Tool.

BACK‐on‐LINE total mean score was 31.9 (SD 12.6). Mean disability was low to moderate (RMDQ = 6.2, SD 5.0), and most participants reported mild to moderate pain intensity (NPRS) and were classified as low to medium risk on the SBST.

### 3.2 Reliability

#### 3.2.1 Internal Consistency

The 34‐item BACK‐on‐LINE showed strong internal consistency (Cronbach's *α* = 0.83). Subdomains PB, PL and PP demonstrated strong to very strong correlations with the total score, whereas PW showed a weaker association (Table 3). All domain‐total correlations were statistically significant (*p* < 0.001).

**TABLE 3:** Internal consistency and domain‐total correlations of BACK‐on‐LINE.

Domain	Number of items	Domain‐to‐total correlation (*r*)	Cronbach's alpha (*α*)
BACK‐on‐LINE total	34	—	0.83
PB	18	0.96	—
PL	3	0.64	—
PP	7	0.75	—
PW	6	0.31	—

*Note:* All domain‐to‐total correlations are statistically significant (*p* < 0.001). The Cronbach's alpha value is reported for the total BACK‐on‐LINE scale only.

Abbreviations: PB, Pain behaviour; PL, Impact of LBP on life; PP, Perception of Pain; PW, Impact of LBP on work.

#### 3.2.2 Test–Retest Reliability

BACK‐on‐LINE showed high test–retest reliability over 4 weeks (ICC = 0.88; 95% CI: 0.78–0.94). Among its domains, PB demonstrated excellent stability (ICC = 0.91, 95% CI: 0.83–0.95), PP good stability (ICC = 0.63, 95% CI: 0.38–0.80), and PL (ICC = 0.56, 95% CI: 0.28–0.76) and PW (ICC = 0.41, 95% CI: 0.08–0.65) moderate stability.

### 3.3 Validity

#### 3.3.1 Convergent Validity

Table 4 presents correlations supporting BACK‐on‐LINE^’^s convergent validity. The total BACK‐on‐LINE score demonstrated a strong correlation with SBST (*r* = 0.67, *p* < 0.001) and RMDQ (*r* = 0.61, *p* < 0.001), and moderate correlation with NPRS (*r* = 0.47, *p* < 0.001). At the subdomain level, PB correlated strongly with SBST (*r* = 0.58) and RMDQ (*r* = 0.56), and a moderately with NPRS (*r* = 0.46), all *p* < 0.001. PL showed strong correlations with RMDQ (*r* = 0.59) and SBST (*r* = 0.55), and a weaker correlation with NPRS (*r* = 0.38), all significant at *p* < 0.001. PP demonstrated a strong correlation with SBST (*r* = 0.60), moderate correlation with RMDQ (*r* = 0.44), and a weaker but significant correlation with NPRS (*r* = 0.31). In contrast, PW displayed weak correlations with all measures, with only the correlation with SBST reaching significance (*r* = 0.18, *p* = 0.037).

**TABLE 4:** Convergent Validity; Correlations of BACK‐on‐LINE total and subdomain scores with other patient‐reported outcome measures (PROMs).

Variables	NPRS	RMDQ	SBST
BACK‐on‐LINE total	*r* = 0.47, *p* < 0.001	*r* = 0.61, *p* < 0.001	*r* = 0.67, *p* < 0.001
PB	*r* = 0.46, *p* < 0.001	*r* = 0.56, *p* < 0.001	*r* = 0.58, *p* < 0.001
PL	*r* = 0.38, *p* < 0.001	*r* = 0.59, *p* < 0.001	*r* = 0.55, *p* < 0.001
*P*P	*r* = 0.31, *p* < 0.001	*r* = 0.44, *p* < 0.001	*r* = 0.60, *p* < 0.001
PW	r = −0.01, *p* = 0.968	*r* = 0.10, *p* = 0.257	*r* = 0.18, *p* = 0.037

Abbreviations: NPRS, Numeric Pain Rating Scale; PB, Pain behaviour; PL, Impact of LBP on life; PP, Perception of Pain; PW, Impact of LBP on work; RMDQ, Roland‐Morris Disability Questionnaire; SBST, STarT Back Screening Tool.

#### 3.3.2 Known‐Groups Validity

According to BACK‐on‐LINE classification, 93 participants (68.4%) were classified as having nociceptive pain and 43 (31.6%) as having nociplastic pain. The nociplastic group had a significantly higher mean total BACK‐on‐LINE score compared to the nociceptive group (46.81 vs. 25.00; *p* < 0.001). A Wilcoxon Signed Rank test showed significantly greater disability (RMDQ) scores in the nociplastic group (9.93 ± 5.88) than in the nociceptive group (4.47 ± 3.45; *p* < 0.001). NPRS and SBST category distributions also differed significantly between groups (*p* < 0.001). Effect size analyses indicated strong known‐groups validity for BACK‐on‐LINE, with large effects for the total score and for key domains (PB, PL, PP), as well as for RMDQ. Comparing scores across each BACK‐on‐LINE domain confirmed that subgroups differed significantly across all constructs measured, supporting the tool's ability to distinguish between pain presentations based on hypothesised clinical profiles (Table 5).

**TABLE 5:** Known‐Groups Validity: Differences in BACK‐on‐LINE total and subdomain scores and other patient‐reported outcomes between nociceptive and nociplastic pain subgroups.

Variable, mean score (SD)	Nociceptive pain (*N* = 93)	Nociplastic pain (*N* = 43)	*p*	Effect size (95% CI)
**BACK‐on‐LINE total**	25.0 (6.28)	46.81 (9.43)	< 0.001	2.94 (2.44–3.45)
PB	14.92 (4.44)	30.42 (7.71)	< 0.001	2.73 (2.24–3.22)
PW	1.87 (1.39)	2.51 (1.56)	0.017	0.44 (0.08–0.81)
PP	6.05 (2.30)	9.63 (1.81)	< 0.001	1.65 (1.24–2.06)
PL	2.15 (1.89)	4.26 (1.67)	< 0.001	1.16 (0.77–1.54)
**RMDQ**	4.47 (3.45)	9.93 (5.88)	< 0.001	1.25 (0.86–1.64)
**SBST, *n* (%)**				
Low risk	77 (82.8%)	8 (18.6%)	< 0.001	0.63 (0.50–0.76)
Medium risk	15 (16.1%)	27 (62.8%)		
High risk	1 (1.1%)	8 (18.6%)		
**NPRS, *n* (%)**				
0–3	35 (37.6%)	3 (7.0%)	< 0.001	0.38 (0.24–0.52)
4–6	45 (48.4%)	22 (51.2%)		
7–10	13 (14.0%)	18 (41.9%)		

Abbreviations: NPRS, Numeric Pain Rating Scale; PB, Pain behaviour; PL, Impact of LBP on life; PP, Perception of Pain; PW, Impact of LBP on work; RMDQ, Roland‐Morris Disability Questionnaire; SBST, STarT Back Screening Tool.

#### 3.3.3 Criterion Validity

To evaluate criterion validity, BACK‐on‐LINE classification performance against established clinical reference standards for high‐risk disabling LBP was examined (Table 6). BACK‐on‐LINE demonstrated low‐to‐moderate criterion validity (AUC = 0.67–0.77) and showed comparable, and in some domains higher, discriminatory performance than the SBST within this sample and against these predefined thresholds.

**TABLE 6:** Criterion validity of BACK‐on‐LINE compared to the STarT Back Screening Tool (SBST) using established clinical reference standards.

Reference standard	BACK‐on‐LINE AUC (95% CI)	SBST AUC (95% CI)	*p*
Pain intensity (NPRS ≥ 7)	0.67 (0.57–0.77)	0.54 (0.48–0.60)	0.017
Disability (RMDQ ≥ 14)	0.77 (0.66–0.88)	0.54 (0.45–0.63)	< 0.001
Pain duration (≥ 6 months)	0.68 (0.60–0.76)	0.53 (0.48–0.57)	< 0.001
Sickness absence (≥ 4 weeks)	0.71 (0.60–0.82)	0.52 (0.45–0.58)	0.001

Abbreviations: AUC, area under the receiver operating characteristic curve; NPRS, Numeric Pain Rating Scale; RMDQ, Roland‐Morris Disability Questionnaire.

## 4 Discussion

This study evaluated the reliability and validity of BACK‐on‐LINE, a self‐administered digital pain phenotyping tool designed to stratify low back pain (LBP) into nociceptive and nociplastic presentations in working adults. By examining reliability and multiple facets of validity against established patient‐reported outcome measures (PROMs), we aimed to determine whether BACK‐on‐LINE provides a robust and scalable approach to identifying dominant pain phenotypes in workplace settings. Unlike approaches that rely on specialist assessment, BACK‐on‐LINE enables individuals to self‐classify their pain based on clinically informed features, supporting earlier, tailored self‐management at the point of need.

### 4.1 BACK‐on‐LINE Reliability

BACK‐on‐LINE demonstrated strong internal consistency and high test–retest reliability, indicating good item coherence and stability of self‐classification over time. These values are comparable to established multidimensional LBP instruments such as the SBST, Oswestry Disability Index (ODI) and RMDQ (Brouwer et al. 2004; Hill et al. 2008; Koivunen et al. 2024). For multidomain tools, strong internal consistency suggests that domains collectively capture a coherent construct without excessive redundancy (McCrae et al. 2011).

High test–retest reliability results are consistent with other validated multidimensional pain measures, such as the Brief Pain Inventory (Gandløse et al. 2025), and the McGill Pain Questionnaire (Grafton et al. 2005), reflecting relative stability of key pain characteristics over short periods in chronic musculoskeletal conditions (Cao et al. 2024). In contrast to single‐item intensity measures such as the NPRS, which are sensitive to short‐term fluctuations, BACK‐on‐LINE integrates sensory, behavioural and cognitive–affective features, supporting more stable phenotyping (Calixtre et al. 2021; Fillingim et al. 2016).

The Impact on Work (PW) domain showed lower stability and moderate domain‐total correlations. This likely reflects the dynamic nature of work‐related pain, influenced by changing physical demands, psychosocial stressors, job security and workplace support (Sarallah et al. 2016; Bergen and Labonté 2020; Shockey et al. 2024; Yang et al. 2023). While psychometric strength was lower than other domains, PW remains conceptually central to BACK‐on‐LINE, capturing the lived experience of managing LBP at work. Further refinement and contextual validation of this domain are warranted.

### 4.2 BACK‐on‐LINE Validity

BACK‐on‐LINE demonstrated strong convergent validity, showing moderate‐to‐strong correlations with pain intensity (NPRS), disability (RMDQ) and prognostic risk (SBST). The strongest association with SBST reflects shared psychosocial constructs. Subdomain correlations followed theoretical expectations: pain behaviour aligned with NPRS, impact on life with RMDQ and perceptions of LBP with SBST, supporting construct validity while extending beyond existing measures through explicit pain phenotype stratification.

Known‐groups validity was robust, with individuals classified as nociplastic reporting higher disability and prognostic risk than those classified as nociceptive. Criterion validity was moderate (AUC 0.67–0.77), indicating the tool's ability to discriminate cases of high pain, disability, chronicity and sickness absence, established markers of poor prognosis and reduced workability (Côté et al. 2008; Landmark et al. 2024).

Criterion validity was moderate for key reference standards, indicating meaningful but non‐diagnostic discrimination. Higher AUC values than SBST were observed in this cohort, likely reflecting differences in design rather than overall superiority. SBST was developed for primary care prognostic use, whereas BACK‐on‐LINE incorporates workplace‐relevant and pain phenotype–specific constructs in a population not yet engaged with primary care. The low proportion of SBST high‐risk cases (6.6%) may have further limited its discriminative performance. These findings support the potential utility of BACK‐on‐LINE in workplace settings but require confirmation in larger and more diverse samples.

### 4.3 Clinical Implications

BACK‐on‐LINE demonstrates good reliability and acceptable validity for distinguishing nociceptive and nociplastic pain in working adults. The tool is not intended to replace clinical diagnosis, but to support earlier, more tailored self‐management by addressing a key limitation of current care: delayed, generic interventions that do not reflect LBP heterogeneity (Steinmetz 2022). By enabling rapid, scalable pain phenotype profiling, BACK‐on‐LINE has the potential to support mechanism‐informed self‐management within workplace settings.

From a clinical perspective, identifying whether symptoms more closely align with nociceptive or nociplastic mechanisms can guide more appropriate early strategies. Individuals with predominantly nociceptive pain may benefit from ‘bottom‐up’ approaches such as focus on tissue protection and recovery, activity modification, ergonomic advice and symptom‐guided exercise, while those with nociplastic pain may respond better to ‘top‐down’ strategies including pain neuroscience education, graded activity, pacing, mindfulness and cognitive‐behavioural approaches (Nijs et al. 2023; Kaplan et al. 2024). Many of these strategies can be delivered through supported self‐management when clinically appropriate.

### 4.4 Limitations

This study has several limitations. First, as a self‐administered tool, BACK‐on‐LINE relies on self‐report and may be influenced by response bias related to workplace pressures, stigma or job security concerns (Latkin et al. 2017). This is particularly relevant to items such as ‘I believe that my job caused/contributed to my back pain’ and ‘I feel supported by my line manager and/or co‐workers’. Anonymity and confidentiality were used to mitigate this risk (Larson 2019). Second, the current prototype differentiates nociceptive and nociplastic pain, excluding neuropathic pain as a pragmatic design decision for early self‐use (Bates et al. 2019; Kerckhove et al. 2021). Lastly, attrition at the 4‐week follow‐up limited assessment of responsiveness and longer‐term predictive validity.

### 4.5 Future Research

Future research should focus on four key priorities. First, item reduction is needed to minimise user burden while preserving classification accuracy and reliability. Second, in settings with appropriate clinical oversight, future iterations should explore the feasibility of incorporating a third pain phenotype (e.g., neuropathic pain) without compromising usability or the tool's non‐diagnostic intent. Third, longitudinal studies are required to establish predictive validity for longer‐term outcomes, including persistent pain, disability, sickness absence and work participation. Finally, research should evaluate whether pain phenotype–informed self‐management improves engagement, impacts on clinician‐patient communication and clinical outcomes compared with generic self‐management approaches, particularly when delivered through digital workplace platforms.

## 5 Conclusion

In working adults with low back pain, BACK‐on‐LINE demonstrated good reliability and acceptable multi‐faceted validity for stratifying dominant pain phenotypes. The tool differentiated nociceptive and nociplastic presentations in ways that aligned with established patterns of pain, disability and psychosocial risk, supporting its construct validity. While BACK‐on‐LINE is not intended as a diagnostic instrument, it supports early, mechanism‐informed self‐classification to guide tailored self‐management. By enabling timely, phenotype‐informed insight outside traditional clinical encounters, the tool offers a pragmatic approach to addressing the limitations of generic early advice within workplace and occupational health pathways.

## Author Contributions

M.C.: Designed the study, processed and analysed the data, prepared tables and drafted the initial manuscript. V.S.: Provided senior supervision, offered methodological and analytical guidance, assisted in interpreting the findings and critically reviewed and provided feedback on manuscript drafts. L.S.: Assumed the overall leadership, conceived the study, secured ethical approvals, coordinated participant recruitment and data collection across workplace sites, guided the data analysis and interpretation, and conducted subsequent revision and finalizing the manuscript.

## Funding

This work was sponsored by the China Scholarship Council (grant number 202008060334) and Work and Health Unit Challenge Fund, Department of Work and Pensions, Grant number: CF_100223.

## Conflicts of Interest

The authors declare no conflicts of interest.

## Supporting information


**Data S1:** ejp70268‐sup‐0001‐DataS1.pdf.

**Data S2:** ejp70268‐sup‐0002‐DataS2.docx.

## References

[ejp70268-bib-0001] Alghadir, A. H. , S. Anwer , A. Iqbal , and Z. A. Iqbal . 2018. “Test–Retest Reliability, Validity, and Minimum Detectable Change of Visual Analog, Numerical Rating, and Verbal Rating Scales for Measurement of Osteoarthritic Knee Pain.” Journal of Pain Research 11: 851–856. 10.2147/JPR.S158847. 29731662 PMC5927184

[ejp70268-bib-0002] Alothman, D. , L. Sheeran , and V. Sparkes . 2018. “Development of a Patient Self‐Assessment and Self‐Management Online Tool (BACKonLINE): Part 1—A Delphi Study.” Orthopaedic Proceedings 100‐B, no. SUPP_2: 10. 10.1302/1358-992x.2018.2.010.

[ejp70268-bib-0003] Alothman, D. , L. Sheeran , and V. Sparkes . 2019. “Development of a Patient Self‐Assessment and Self‐Management Online Tool (BACKonLINE): Measurement Properties.” Orthopaedic Proceedings 101‐B, no. SUPP_10: 5. 10.1302/1358-992x.2019.10.005.

[ejp70268-bib-0004] Bates, D. , B. C. Schultheis , M. C. Hanes , et al. 2019. “A Comprehensive Algorithm for Management of Neuropathic Pain.” Pain Medicine 20, no. Suppl 1: S2–s12. 10.1093/pm/pnz075. PMC6544553 31152178

[ejp70268-bib-0005] Bergen, N. , and R. Labonté . 2020. “‘Everything Is Perfect, and we Have no Problems’: Detecting and Limiting Social Desirability Bias in Qualitative Research.” Qualitative Health Research 30, no. 5: 783–792. 10.1177/1049732319889354. 31830860

[ejp70268-bib-0006] Bittencourt, J. V. , M. C. Bezerra , M. R. Pina , F. J. J. Reis , A. de Sá Ferreira , and L. A. C. Nogueira . 2022. “Use of the painDETECT to Discriminate Musculoskeletal Pain Phenotypes.” Archives of Physiotherapy 12, no. 1: 7. 10.1186/s40945-022-00129-2. 35172904 PMC8851806

[ejp70268-bib-0007] Boonstra, A. M. , R. E. Stewart , A. J. Köke , et al. 2016. “Cut‐Off Points for Mild, Moderate, and Severe Pain on the Numeric Rating Scale for Pain in Patients With Chronic Musculoskeletal Pain: Variability and Influence of Sex and Catastrophizing.” Frontiers in Psychology 7: 1466. 10.3389/fpsyg.2016.01466. 27746750 PMC5043012

[ejp70268-bib-0008] Bouhassira, D. , N. Attal , H. Alchaar , et al. 2005. “Comparison of Pain Syndromes Associated With Nervous or Somatic Lesions and Development of a New Neuropathic Pain Diagnostic Questionnaire (DN4).” Pain 114, no. 1–2: 29–36. 10.1016/j.pain.2004.12.010. 15733628

[ejp70268-bib-0009] Brouwer, S. , W. Kuijer , P. U. Dijkstra , L. N. Göeken , J. W. Groothoff , and J. H. Geertzen . 2004. “Reliability and Stability of the Roland Morris Disability Questionnaire: Intra Class Correlation and Limits of Agreement.” Disability and Rehabilitation 26, no. 3: 162–165. 10.1080/09638280310001639713. 14754627

[ejp70268-bib-0010] Bruyère, O. , M. Demoulin , C. Beaudart , et al. 2014. “Validity and Reliability of the French Version of the STarT Back Screening Tool for Patients With Low Back Pain.” Spine 39, no. 2: E123–E128. 10.1097/BRS.0000000000000062. 24108286

[ejp70268-bib-0011] Calixtre, L. B. , C. L. Fonseca , B. Gruninger , and D. H. Kamonseki . 2021. “Psychometric Properties of the Brazilian Version of the Bournemouth Questionnaire for Low Back Pain: Validity and Reliability.” Brazilian Journal of Physical Therapy 25, no. 1: 70–77. 10.1016/j.bjpt.2020.02.003. 32151526 PMC7817867

[ejp70268-bib-0012] Cao, B. , Q. Xu , Y. Shi , et al. 2024. “Pathology of Pain and Its Implications for Therapeutic Interventions.” Signal Transduction and Targeted Therapy 9, no. 1: 155. 10.1038/s41392-024-01845-w. 38851750 PMC11162504

[ejp70268-bib-0013] Chen, M. , V. Sparkes , and L. Sheeran . 2023. “Patient Self‐Assessment Tool (Back‐on‐Line) for Early Targeted Self‐Management of Low Back Pain in the Workplace: Construct and Discriminate Validation Study [PO‐1‐078] 2023 World Physiotherapy, Dubai, UAE.” https://world.physio/congress‐proceeding/patient‐self‐assessment‐tool‐back‐linetm‐early‐targeted‐self‐management‐low.

[ejp70268-bib-0014] Chen, M. , V. Sparkes , and L. Sheeran . 2025. “Systematic Review of Digital Health Interventions to Support Self‐Management of Low Back Pain in the Workplace.” DIGITAL HEALTH 11: 20552076251336281. 10.1177/20552076251336281. 40438190 PMC12117239

[ejp70268-bib-0015] Chimenti, R. L. , L. A. Frey‐Law , and K. A. Sluka . 2018. “A Mechanism‐Based Approach to Physical Therapist Management of Pain.” Physical Therapy 98, no. 5: 302–314. 10.1093/ptj/pzy030. 29669091 PMC6256939

[ejp70268-bib-0016] Côté, P. , M. L. Baldwin , W. G. Johnson , J. W. Frank , and R. J. Butler . 2008. “Patterns of Sick‐Leave and Health Outcomes in Injured Workers With Back Pain.” European Spine Journal 17: 484–493. 10.1007/s00586-007-0577-6. 18214554 PMC2295281

[ejp70268-bib-0017] Dubé, M. O. , P. Langevin , and J. S. Roy . 2021. “Measurement Properties of the Pain Self‐Efficacy Questionnaire in Populations With Musculoskeletal Disorders: A Systematic Review.” PAIN Reports 6, no. 4: e972. 10.1097/pr9.0000000000000972. 34963996 PMC8701870

[ejp70268-bib-0018] Eton, D. T. , M. K. Lee , J. L. St. Sauver , and R. T. Anderson . 2020. “Known‐Groups Validity and Responsiveness to Change of the Patient Experience With Treatment and Self‐Management (PETS vs. 2.0): A Patient‐Reported Measure of Treatment Burden.” Quality of Life Research 29: 3143–3154. 10.1007/s11136-020-02546-x. 32524346 PMC8012109

[ejp70268-bib-0019] Fatoye, F. , T. Gebrye , and I. Odeyemi . 2019. “Real‐World Incidence and Prevalence of Low Back Pain Using Routinely Collected Data.” Rheumatology International 39: 619–626. 10.1007/s00296-019-04273-0. 30848349

[ejp70268-bib-0020] Ferreira, M. L. , K. de Luca , L. M. Haile , et al. 2023. “Global, Regional, and National Burden of Low Back Pain, 1990–2020, Its Attributable Risk Factors, and Projections to 2050: A Systematic Analysis of the Global Burden of Disease Study 2021.” Lancet Rheumatology 5, no. 6: e316–e329. 10.1016/S2665-9913(23)00098-X. 37273833 PMC10234592

[ejp70268-bib-0021] Fillingim, R. B. , J. D. Loeser , R. Baron , and R. R. Edwards . 2016. “Assessment of Chronic Pain: Domains, Methods, and Mechanisms.” Journal of Pain 17, no. 9: T10–T20. 10.1016/j.jpain.2015.08.010. 27586827 PMC5010652

[ejp70268-bib-0022] Fuhro, F. F. , F. R. C. Fagundes , A. C. T. Manzoni , L. O. P. Costa , and C. M. N. Cabral . 2016. “Örebro Musculoskeletal Pain Screening Questionnaire Short‐Form and STarT Back Screening Tool: Correlation and Agreement Analysis.” Spine 41, no. 15: E931–E936. 10.1097/BRS.0000000000001415. 26720177

[ejp70268-bib-0023] Gandløse, J. S. , S. W. M. Christensen , D. F. Lambertsen , Ó. E. Árnason , J. Vela , and T. S. Palsson . 2025. “Validity and Reliability of the Danish Version of the Short Form Brief Pain Inventory.” Musculoskeletal Science & Practice 75: 103242. 10.1016/j.msksp.2024.103242. 39637831

[ejp70268-bib-0024] George, D. 2011. SPSS for Windows Step by Step: A Simple Study Guide and Reference, 17.0 Update, 10/e. Pearson Education India.

[ejp70268-bib-0026] Grafton, K. V. , N. E. Foster , and C. C. Wright . 2005. “Test‐Retest Reliability of the Short‐Form McGill Pain Questionnaire: Assessment of Intraclass Correlation Coefficients and Limits of Agreement in Patients With Osteoarthritis.” Clinical Journal of Pain 21, no. 1: 73–82. 10.1097/00002508-200501000-00009. 15599134

[ejp70268-bib-0027] Hill, J. C. , K. M. Dunn , M. Lewis , et al. 2008. “A Primary Care Back Pain Screening Tool: Identifying Patient Subgroups for Initial Treatment.” Arthritis Care & Research: Official Journal of the American College of Rheumatology 59, no. 5: 632–641. 10.1002/art.23563. 18438893

[ejp70268-bib-0029] Hill, J. C. , D. G. Whitehurst , M. Lewis , et al. 2011. “Comparison of Stratified Primary Care Management for Low Back Pain With Current Best Practice (STarT Back): A Randomised Controlled Trial.” Lancet 378, no. 9802: 1560–1571. 10.1016/S0140-6736(11)60937-9. 21963002 PMC3208163

[ejp70268-bib-0031] Jenks, A. , T. Hoekstra , M. van Tulder , R. W. Ostelo , S. M. Rubinstein , and A. Chiarotto . 2022. “Roland‐Morris Disability Questionnaire, Oswestry Disability Index, and Quebec Back Pain Disability Scale: Which Has Superior Measurement Properties in Older Adults With Low Back Pain?” Journal of Orthopaedic & Sports Physical Therapy 52, no. 7: 457–469. 10.2519/jospt.2022.10802. 35584027

[ejp70268-bib-0032] Kahl, C. , and J. A. Cleland . 2005. “Visual Analogue Scale, Numeric Pain Rating Scale and the McGill Pain Questionnaire: An Overview of Psychometric Properties.” Physical Therapy Reviews 10, no. 2: 123–128. 10.1179/108331905X55776.

[ejp70268-bib-0033] Kaplan, C. M. , E. Kelleher , A. Irani , A. Schrepf , D. J. Clauw , and S. E. Harte . 2024. “Deciphering Nociplastic Pain: Clinical Features, Risk Factors and Potential Mechanisms.” Nature Reviews Neurology 20: 1–17. 10.1038/s41582-024-00966-8. 38755449

[ejp70268-bib-0034] Kerckhove, N. , C. Lambert , A. Corteval , B. Pereira , A. Eschalier , and C. Dualé . 2021. “Cross‐Sectional Study of Prevalence, Characterization and Impact of Chronic Pain Disorders in Workers.” Journal of Pain 22, no. 5: 520–532. 10.1016/j.jpain.2020.11.005. 33309785

[ejp70268-bib-0035] Kim, H.‐Y. 2017. “Statistical Notes for Clinical Researchers: Chi‐Squared Test and Fisher's Exact Test.” Restorative Dentistry & Endodontics 42, no. 2: 152–155. 10.5395/rde.2017.42.2.152. 28503482 PMC5426219

[ejp70268-bib-0036] Koivunen, K. , S. Widbom‐Kolhanen , K. Pernaa , J. Arokoski , and M. Saltychev . 2024. “Reliability and Validity of Oswestry Disability Index Among Patients Undergoing Lumbar Spinal Surgery.” BMC Surgery 24, no. 1: 13. 10.1186/s12893-023-02307-w. 38172794 PMC10765861

[ejp70268-bib-0037] Landmark, L. , H. F. Sunde , E. A. Fors , et al. 2024. “Associations Between Pain Intensity, Psychosocial Factors, and Pain‐Related Disability in 4285 Patients With Chronic Pain.” Scientific Reports 14, no. 1: 13477. 10.1038/s41598-024-64059-8. 38866885 PMC11169509

[ejp70268-bib-0038] Larson, R. B. 2019. “Controlling Social Desirability Bias.” International Journal of Market Research 61, no. 5: 534–547. 10.1177/1470785318805305.

[ejp70268-bib-0039] Latkin, C. A. , C. Edwards , M. A. Davey‐Rothwell , and K. E. Tobin . 2017. “The Relationship Between Social Desirability Bias and Self‐Reports of Health, Substance Use, and Social Network Factors Among Urban Substance Users in Baltimore, Maryland.” Addictive Behaviors 73: 133–136. 10.1016/j.addbeh.2017.05.005. 28511097 PMC5519338

[ejp70268-bib-0040] Mandrekar, J. N. 2010. “Receiver Operating Characteristic Curve in Diagnostic Test Assessment.” Journal of Thoracic Oncology 5, no. 9: 1315–1316. 10.1097/JTO.0b013e3181ec173d. 20736804

[ejp70268-bib-0041] Mason, S. J. , L. M. Brading , K. Kane , P. G. Conaghan , S. R. Kingsbury , and G. A. McHugh . 2024. “Barriers and Facilitators to Engaging With a Digital Self‐Management Programme for Painful Distal Upper Limb Musculoskeletal Disorders: A Qualitative Exploratory Study.” Health Expectations 27, no. 3: e14056. 10.1111/hex.14056. 38858844 PMC11164711

[ejp70268-bib-0042] McCrae, R. R. , J. E. Kurtz , S. Yamagata , and A. Terracciano . 2011. “Internal Consistency, Retest Reliability, and Their Implications for Personality Scale Validity.” Personality and Social Psychology Review 15, no. 1: 28–50. 10.1177/1088868310366253. 20435807 PMC2927808

[ejp70268-bib-0043] NICE . 2016. National Institute for Health and Care Excellence Guidelines: Low Back Pain and Sciatica in Over 16s: Assessment and Management. NICE. https://www.nice.org.uk/guidance/ng59. 27929617

[ejp70268-bib-0045] Nicholl, B. I. , L. F. Sandal , M. J. Stochkendahl , et al. 2017. “Digital Support Interventions for the Self‐Management of Low Back Pain: A Systematic Review.” Journal of Medical Internet Research 19, no. 5: e179. 10.2196/jmir.7290. 28550009 PMC5466697

[ejp70268-bib-0046] Nijs, J. , L. De Baets , and P. Hodges . 2023. “Phenotyping Nociceptive, Neuropathic, and Nociplastic Pain: Who, How, & Why?” Brazilian Journal of Physical Therapy 27, no. 4: 100537. 10.1016/j.bjpt.2023.100537. 37639943 PMC10470273

[ejp70268-bib-0047] Nijs, J. , E. Kosek , A. Chiarotto , et al. 2024. “Nociceptive, Neuropathic, or Nociplastic Low Back Pain? The Low Back Pain Phenotyping (BACPAP) Consortium's International and Multidisciplinary Consensus Recommendations.” Lancet Rheumatology 6, no. 3: e178–e188. 10.1016/S2665-9913(23)00324-7. 38310923

[ejp70268-bib-0048] Portney, L. G. , and M. P. Watkins . 2009. Foundations of Clinical Research: Applications to Practice. Vol. 892. Pearson/Prentice Hall Upper Saddle River.

[ejp70268-bib-0049] Roland, M. , and R. Morris . 1983. “A Study of the Natural History of Back Pain. Part I: Development of a Reliable and Sensitive Measure of Disability in Low‐Back Pain.” Spine (Phila Pa 1976) 8, no. 2: 141–144. 10.1097/00007632-198303000-00004. 6222486

[ejp70268-bib-0050] Sarallah, S. , T. S. Sadat , A. R. Jamshidi , and W. Joan . 2016. “A Multidisciplinary Work‐Related Low Back Pain Predictor Questionnaire: Psychometric Evaluation of Iranian Patient‐Care Workers.” Asian Spine Journal 10, no. 3: 501–508. 10.4184/asj.2016.10.3.501. 27340530 PMC4917769

[ejp70268-bib-0051] Scholz, C. , P. Schmigalle , C. Plessen , et al. 2024. “The Effect of Self‐Management Techniques on Relevant Outcomes in Chronic Low Back Pain: A Systematic Review and Meta‐Analysis.” European Journal of Pain 28, no. 4: 532–550. 10.1002/ejp.2221. 38071425

[ejp70268-bib-0052] Shockey, T. , T. Alterman , H. Yang , and M. L. Lu . 2024. “Workplace Psychosocial Factors, Work Organization, and Physical Exertion as Risk Factors for Low Back Pain Among US Workers: Data From the 2015 National Health Interview Survey.” Journal of Occupational and Environmental Medicine 66, no. 6: 467–474. 10.1097/jom.0000000000003087. 38471812 PMC11683765

[ejp70268-bib-0053] Smart, K. M. , C. Blake , A. Staines , and C. Doody . 2011. “The Discriminative Validity of ‘Nociceptive,’ ‘Peripheral Neuropathic,’ and ‘Central Sensitization’ as Mechanisms‐Based Classifications of Musculoskeletal Pain.” Clinical Journal of Pain 27, no. 8: 655–663. 10.1097/AJP.0b013e318215f16a. 21471812

[ejp70268-bib-0054] Smart, K. M. , C. Blake , A. Staines , M. Thacker , and C. Doody . 2012. “Mechanisms‐Based Classifications of Musculoskeletal Pain: Part 3 of 3: Symptoms and Signs of Nociceptive Pain in Patients With Low Back (±Leg) Pain.” Manual Therapy 17, no. 4: 352–357. 10.1016/j.math.2012.03.002. 22464885

[ejp70268-bib-0055] Smart, K. M. , A. Curley , C. Blake , A. Staines , and C. Doody . 2010. “The Reliability of Clinical Judgments and Criteria Associated With Mechanisms‐Based Classifications of Pain in Patients With Low Back Pain Disorders: A Preliminary Reliability Study.” Journal of Manual & Manipulative Therapy 18, no. 2: 102–110. 10.1179/106698110X12640740712897. 21655393 PMC3101074

[ejp70268-bib-0056] Steinmetz, A. 2022. “Back Pain Treatment: A New Perspective.” Therapeutic Advances in Musculoskeletal Disease 14: 1–13. 10.1177/1759720X221100293. PMC9260567 35814351

[ejp70268-bib-0057] Stratford, P. W. , and D. L. Riddle . 2016. “A Roland Morris Disability Questionnaire Target Value to Distinguish Between Functional and Dysfunctional States in People With Low Back Pain.” Physiotherapy Canada 68, no. 1: 29–35. 10.3138/ptc.2014-85. 27504045 PMC4961316

[ejp70268-bib-0060] Wang, E. , I. B. Rodrigues , and L. C. Li . 2024. “Effectiveness of Tailored Self‐Management Interventions for People With Chronic Musculoskeletal Conditions: A Systematic Review and Meta‐Analysis.” Physiotherapy Canada 77, no. 2: 148–161. 10.3138/ptc-2023-0050. PMC13167186 42130691

[ejp70268-bib-0061] WHO . 2001. International Classification of Functioning, Disability and Health (ICF). World Health Organization. https://www.who.int/standards/classifications/international‐classification‐of‐functioning‐disability‐and‐health.

[ejp70268-bib-0062] WHO . 2023. World Health Organization Guideline for Non‐Surgical Management of Chronic Primary Low Back Pain in Adults in Primary and Community Care Settings. Guidelines Review Committee, Geneva World Health Organization. https://www.who.int/publications/i/item/9789240081789. 38198579

[ejp70268-bib-0063] Yang, H. , M. L. Lu , S. Haldeman , and N. Swanson . 2023. “Psychosocial Risk Factors for Low Back Pain in US Workers: Data From the 2002–2018 Quality of Work Life Survey.” American Journal of Industrial Medicine 66, no. 1: 41–53. 10.1002/ajim.23444. 36420950 PMC10123870

