# Supplementary material for: BACK‐on‐LINE™ : A Digital Pain Phenotyping Tool to Personalise Early Self‐Management of Low Back Pain: Reliability and Validity in Working Adults
Source: Eur J Pain. 2026 Apr 11;30(4):e70268. doi: 10.1002/ejp.70268 (PMC13069916; doi:10.1002/ejp.70268)
Supplement: Supplementary file 1 — Data S1: ejp70268‐sup‐0001‐DataS1.pdf. [file EJP-30-0-s001.pdf]

1. About my back pain

1.1 Do you know what caused your current back pain?

Yes (0)

No (2)

Not sure (1)

1.2 How long have you had this current episode of back pain?

0-7 days (0)

8-14 days (0)

15 days to 1 month (0)

More than 1 month (0)

More than 3 months (1)

More than 6 months (2)

1.3 On the scale below where 0 means no pain, and 10 means worst possible pain

How intense was your pain on average **last week**?

Sliding scale 0-10

Scoring rule (0-3) (0), 4-6 (1), 7-10 (2)

1.4 If you have been treated for back pain, were you satisfied with your treatment?

Yes, I was satisfied with the treatment (0)

I was neither satisfied nor dissatisfied with the treatment (1)

No, I was not satisfied with the treatment (2)

I was never treated for back pain (0)

1.5_Branch Do you currently take medication to manage your back pain?

Yes (Continue to Q1.5)

No (Jump to Q1.7)

1.5 What medication do you currently take to manage your back pain? Please tick **all** that apply.

Paracetamol (0)

Ibuprofen (also known as Nuromol) (1)

Naproxen (2)

Diclofenac (2)

Celecoxib (2)

Codeine/Co-codamol (2)

Tramadol (3)

Amitriptyline (3)

Gabapentin (3)

Pregabalin (also known as Lyrica) (3)

Duloxetine/Cymbalta (3)

Buprenorphine (known as BuTrans, Transtec) (3)

Morphine (also known as Oramorph, MST, Zomorph) (3)

Oxycodone (also known as Longtec, Shorttec) (3)

Fentanyl (patches) (also known as Durogesic or Matrifen) (3)

Other (1)

1.6 How effective is the medication in reducing your back pain?

Effective (0)

Not sure (1)

Ineffective (2)

1.7 Where is your pain? Please tick **all** that apply

Neck (1)

Right shoulder (1)

Left shoulder (1)

Right arm (1)

Left arm (1)

Upper back (1)

Lower back (1)

Right buttock (1)

Left buttock (1)

Right hip (1)

Left hip (1)

Right leg (1)

Left leg (1)

1.8 Is your pain there all the time?

My pain is there all the time (2)

My pain comes and goes (0)

Not sure (1)

1.9 What type of pain is it? Please tick **all** options that apply

Deep (1)

Nagging (1)

Dull (1)

Sharp (1)

Shooting (1)

Dull ache (1)

Like an electric shock (1)

Burning (1)

Pressure (1)

Stinging (1)

Aching (1)

Throbbing (1)

Spread over a wide area (1)

1.10 When is your pain at its worst?

It depends on what I do (0)

In the morning (0)

During the day (0)

At the end of the day (0)

My pain is there all day long (2)

1.11 Is your back pain getting better, staying the same or getting worse?

My pain is getting better (0)

My pain is the same (1)

My pain is getting worse (2

1.12 Can you ease your back pain?

Yes (0)

Sometimes (1)

No (2)

1.13 What eases your back pain? Please tick **all** that apply

I avoid activities that cause me pain (2)

Taking medication (1)

Changing positions (0)

Lying down (0)

Sleep (0)

Walking (0)

Standing (0)

Sitting down (0)

Exercise/stretching (0)

Massage (1)

Hot/cold pack (0)

Other (0)

1.14 What aggravates your back pain? Please tick **all** that apply

Sitting down (0)

Standing (0)

Walking (0)

Lying down (0)

Exercise (2)

Lifting/ carrying load (0)

Forward bending (stooping) (0)

Working on a computer (0)

Housework/Gardening (0)

Any activity that I do for a long period of time increases my back pain (1)

Everything I do causes me pain (2)

1.15 Is this the first time you have experienced this type of pain?

Yes (0)

No (1)

1.16 Other than your back pain, do you experience any other sensations? Please tick **all** that apply

Pins and needles (1)

Numbness (1)

Tingling (1)

Burning (1)

Stinging (1)

Pressure (1)

None of the above (0)

1.17 Does your back pain wake you up at night?

Yes (2)

Sometimes (1)

No (0)

1.18 If you wake up with back pain, can you get back to sleep?

Yes (0)

Sometimes (1)

No (2)

2. **How my back pain impacts on my work**

2.1 I believe that my job caused /contributed to my back pain.

Agree (2)

Neither agree nor disagree (1)

Disagree (0)

Not applicable (0)

2.2 I feel supported by my line manager and/or co-workers,

Agree (0)

Neither agree nor disagree (1)

Disagree (2)

Not applicable (0)

2.3 How much is your back pain affecting your work?

Not at all (0)

Sometimes (0)

Frequently (1)

I am unable to work because of my back pain (2)

Not applicable (0)

2.4 Are you currently off work right now because of your back pain?

Yes (2)

No (0)

2.5 How long have you been off work?

Less than 3 months (1)

Between 3 to 6 months (2)

More than 6 months (3)

2.6 How likely is it that you would return to work within six months?

Very Likely (0)

Likely (1)

Not sure (2)

Unlikely (3)

3. How my back pain impacts on my lifestyle, social and family life

3.1 I can’t do my normal daily activities because of my back pain

agree (2)

neither agree nor disagree (1)

disagree (0)

3.2 My back pain is affecting my social life

agree (2)

neither agree nor disagree (1)

disagree (0)

3.3 My back pain is affecting my relationships with my significant others

agree (2)

neither agree nor disagree (1)

disagree (0)

4. How my back pain impacts on me personally

4.1 My back pain makes me feel stressed/anxious.

agree (2)

neither agree nor disagree (1)

disagree (0)

4.2 Stress increases my back pain.

agree (2)

neither agree nor disagree (1)

disagree (0)

4.3 Being active and exercise increases my back pain.

agree (2)

neither agree nor disagree (1)

disagree (0)

4.4 Since my back pain started, I feel more tired.

agree (2)

neither agree nor disagree (1)

disagree (0)

4.5 Irrespective of my back pain I continue to take interest and/or pleasure in doing things.

agree (0)

neither agree nor disagree (1)

disagree (2)

4.6 My family and friends understand what I'm going through with my back pain.

agree (0)

neither agree nor disagree (1)

disagree (2)

4.7 I am very confident that my back pain will eventually go away.

agree (0)

neither agree nor disagree (1)

disagree (2)
