# Supplementary material for: BACK‐on‐LINE™ : A Digital Pain Phenotyping Tool to Personalise Early Self‐Management of Low Back Pain: Reliability and Validity in Working Adults
Source: Eur J Pain. 2026 Apr 11;30(4):e70268. doi: 10.1002/ejp.70268 (PMC13069916; doi:10.1002/ejp.70268)
Supplement: Supplementary file 2 — Data S2: ejp70268‐sup‐0002‐DataS2.docx. [file EJP-30-0-s002.docx]

**Data S2**

**BACK-on-LINE^TM^ tool scoring direction and its theoretical basis for each item**

| **Domain** | **Item** | **Question (Abbreviated)** | **Response Option (Example)** | **Score** | **Theoretical basis in accordance with The Low Back Pain Phenotyping (BACPAP) consortium's international and multidisciplinary consensus recommendations (Nijs et al 2024)** | **Mechanistic Signal** |
| --- | --- | --- | --- | --- | --- | --- |
| Pain Behaviour (Pattern, Modulation, Aggravating/Easing Factors) | 1.1 | Cause of pain | Yes | 0 | Identifiable peripheral nociceptive input | Nociceptive |
|  |  |  | Not sure | 1 | Uncertain driver; possible altered nociception | Mixed |
|  |  |  | No | 2 | Pain without clear tissue injury | Nociplastic |
|  | 1.2 | Duration | ≤3 months | 0 | Within healing timeframe | Nociceptive |
|  |  |  | >3 months | 1–2 | Persistent beyond expected healing | Nociplastic |
|  | 1.3 | Intensity  (0–10) | 0–3 | 0 | Proportionate nociceptive response | Nociceptive |
|  |  |  | 4–6 | 1 | Moderate amplification | Mixed |
|  |  |  | 7–10 | 2 | Disproportionate intensity | Nociplastic tendency |
|  | 1.4 | Treatment satisfaction | Satisfied | 0 | Adequate peripheral modulation | Nociceptive |
|  |  |  | Neutral | 1 | Partial modulation | Mixed |
|  |  |  | Not satisfied | 2 | Persistent pain despite care | Nociplastic |
|  | 1.5 | Medication type | Paracetamol | 0 | Peripheral modulation | Nociceptive |
|  |  |  | NSAIDs | 1–2 | Anti-inflammatory modulation | Mixed |
|  |  |  | Opioids/neuromodulators | 3 | Centrally acting modulation | Nociplastic |
|  | 1.6 | Medication effective | Effective | 0 | Peripheral responsiveness | Nociceptive |
|  |  |  | Not sure | 1 | Partial effect | Mixed |
|  |  |  | Ineffective | 2 | Reduced peripheral response | Nociplastic |
|  | 1.7 | Pain distribution | Each region | 1 per region | Increasing spread indicates widespread pain | Nociplastic (cumulative) |
|  | 1.8 | Pain constancy | Comes/goes | 0 | Activity-modulated pattern | Nociceptive |
|  |  |  | Not sure | 1 | Partial modulation | Mixed |
|  |  |  | All the time | 2 | Persistent independent of load | Nociplastic |
|  | 1.9 | Pain descriptors | Each descriptor | 1 each | Sensory amplification features | Nociplastic |
|  | 1.10 | When is pain worst | Activity/time dependent | 0 | Context-linked nociception | Nociceptive |
|  |  |  | All day long | 2 | Reduced contextual modulation | Nociplastic |
|  | 1.11 | Pain trajectory | Improving | 0 | Resolution of peripheral input | Nociceptive |
|  |  |  | Same | 1 | Persistent but stable | Mixed |
|  |  |  | Worse | 2 | Escalating altered processing | Nociplastic |
|  | 1.12 | Can ease pain | Yes | 0 | Preserved endogenous inhibition | Nociceptive |
|  |  |  | Sometimes | 1 | Partial inhibition | Mixed |
|  |  |  | No | 2 | Impaired inhibition | Nociplastic |
|  | 1.13 | Easing strategies | Active mvt/position | 0 | Maintained load tolerance | Nociceptive |
|  |  |  | Medication/massage | 1 | External modulation reliance | Mixed |
|  |  |  | Avoidance | 2 | Fear-avoidance behaviour | Nociplastic |
|  | 1.14 | Aggravating factors | Load-specific | 0 | Proportionate mechanical response | Nociceptive |
|  |  |  | Prolonged activity | 1 | Reduced tolerance | Mixed |
|  |  |  | Exercise/everything | 2 | Hypersensitivity to normal activity | Nociplastic |
|  | 1.15 | First episode | Yes | 0 | Acute peripheral input | Nociceptive |
|  |  |  | No | 1 | Recurrence vulnerability | Mixed |
|  | 1.16 | Other sensations | None | 0 | No altered sensory processing | Nociceptive |
|  |  |  | Pins/needles/burning etc. no dermatomal/ myotome pattern | 1 | Altered nociceptive signalling | Nociplastic tendency |
|  | 1.17 | Woken by pain | No | 0 | Load-related pattern | Nociceptive |
|  |  |  | Sometimes | 1 | Partial sleep disturbance | Mixed |
|  |  |  | Yes | 2 | Sleep disturbance linked to central sensitisation | Nociplastic |
|  | 1.18 | Return to sleep | Yes | 0 | Preserved physiological regulation | Nociceptive |
|  |  |  | Sometimes | 1 | Partial dysregulation | Mixed |
|  |  |  | No | 2 | Sustained arousal; altered central processing | Nociplastic |
| Impact on work | 2.1 | Job caused pain | Disagree | 0 | Lower structural attribution | Nociceptive tendency |
|  |  |  | Neutral | 1 | Partial attribution | Mixed |
|  |  |  | Agree | 2 | Persistent threat belief | Nociplastic tendency |
|  | 2.2 | Workplace support | Agree | 0 | Protective psychosocial buffer | Nociceptive tendency |
|  |  |  | Neutral | 1 | Reduced buffer | Mixed |
|  |  |  | Disagree | 2 | Low support; persistence risk | Nociplastic risk |
|  | 2.3 | Work affected | Not at all/sometimes | 0 | Limited interference | Nociceptive |
|  |  |  | Frequently | 1 | Moderate impact | Mixed |
|  |  |  | Unable to work | 2 | Disproportionate functional interference | Nociplastic |
|  | 2.4 | Currently off work | No | 0 | Maintained function | Nociceptive |
|  |  |  | Yes | 2 | Work disability | Nociplastic tendency |
|  | 2.5 | Duration off work | <3 months | 1 | Short persistence | Mixed |
|  |  |  | 3–6 months | 2 | Persistent disability | Nociplastic |
|  |  |  | >6 months | 3 | Long-term disability | Nociplastic |
|  | 2.6 | Likelihood return | Very likely | 0 | Positive recovery expectation | Nociceptive |
|  |  |  | Likely | 1 | Moderate belief | Mixed |
|  |  |  | Not sure | 2 | Uncertain outlook | Nociplastic tendency |
|  |  |  | Unlikely | 3 | Low recovery expectation | Nociplastic |
| Lifestyle, Social & Family Impact | 3.1 | Daily activities affected | Disagree | 0 | Activity-specific limitation | Nociceptive |
|  |  |  | Neutral | 1 | Partial limitation | Mixed |
|  |  |  | Agree | 2 | Widespread interference | Nociplastic |
|  | 3.2 | Social life affected | Disagree | 0 | Limited impact | Nociceptive |
|  |  |  | Neutral | 1 | Moderate impact | Mixed |
|  |  |  | Agree | 2 | Global life interference | Nociplastic |
|  | 3.3 | Relationships affected | Disagree | 0 | Preserved relational function | Nociceptive tendency |
|  |  |  | Neutral | 1 | Partial strain | Mixed |
|  |  |  | Agree | 2 | Interpersonal burden | Nociplastic |
| Personal Impact (Cognitive & Affective Factors) | 4.1 | Pain causes stress/anxiety | Disagree | 0 | Regulated affect | Nociceptive tendency |
|  |  |  | Neutral | 1 | Moderate stress | Mixed |
|  |  |  | Agree | 2 | Affective amplification | Nociplastic |
|  | 4.2 | Stress increases pain | Disagree | 0 | Limited stress–pain facilitation | Nociceptive |
|  |  |  | Neutral | 1 | Partial interaction | Mixed |
|  |  |  | Agree | 2 | Central facilitation pathway | Nociplastic |
|  | 4.3 | Exercise increases pain | Disagree | 0 | Preserved load tolerance | Nociceptive |
|  |  |  | Neutral | 1 | Partial intolerance | Mixed |
|  |  |  | Agree | 2 | Hypersensitivity to activity | Nociplastic |
|  | 4.4 | Increased fatigue | Disagree | 0 | Localised mechanical pattern | Nociceptive |
|  |  |  | Neutral | 1 | Moderate fatigue | Mixed |
|  |  |  | Agree | 2 | Central fatigue component | Nociplastic |
|  | 4.5 | Maintains interest | Agree | 0 | Preserved engagement | Nociceptive tendency |
|  |  |  | Neutral | 1 | Reduced engagement | Mixed |
|  |  |  | Disagree | 2 | Anhedonia associated with centrally modulated pain | Nociplastic |
|  | 4.6 | Social understanding | Agree | 0 | Social buffering | Nociceptive tendency |
|  |  |  | Neutral | 1 | Partial support | Mixed |
|  |  |  | Disagree | 2 | Perceived isolation | Nociplastic risk |
|  | 4.7 | Confidence pain resolves | Agree | 0 | Adaptive recovery belief | Nociceptive |
|  |  |  | Neutral | 1 | Uncertain outlook | Mixed |
|  |  |  | Disagree | 2 | Low recovery expectation; persistence risk | Nociplastic |
